# Supplementary material for: Viral Sequences Detection by High-Throughput Sequencing in Cerebrospinal Fluid of Individuals with and without Central Nervous System Disease
Source: Genes (Basel). 2019 Aug 19;10(8):625. doi: 10.3390/genes10080625 (PMC6723360; doi:10.3390/genes10080625)
Supplement: Supplementary file 1 [file genes-10-00625-s001.pdf]

	Viruses Detected	Sequence (5' to 3')			Final [uM] Fwd/Rev/ Probe	r(RT-)PCR anneling temperature °C	Reference
		Fwd primer	Probe	Rev Primer			
**r(RT-)PCR assays**	Torque Teno virus	GTTTTCYACGCCCGTCC	FAM - ACTCACCTHCGGCACCCGC– BHQ1	CCTTGACTYCGGTGTGTA A	0.9/0.9/0.2	60	Masouridi-Levrat S, et al. Bone Marrow Transplant. 2016;51:440–442.
	Adenovirus	GCCCCAGTGGTCTTACA TGCACATC	FAM - TGCACCAGACCCGGGCTCAGGTACTCCG A –TAMRA	gvGCCACGGTGGGGTTTC TAAACTT	0.9/0.9/0.2	60	**adapted from** Verheyen J, et al.Appl Environ Microbiol 2009; 75: 2798–2801.
	Merkel cell polyomavirus	CCCTTTGGAGCAAATTC CA	FAM - CAAAATATCCACAAGCTCAGAAGTGA – TAMRA	CTGACCTCATCAAACATA GAGAA	0.6/0.6/0.2 5	55	Arvia R. et al. Journal of Virological Methods 246 (2017) 15–20
	Human pegivirus-1	GGCGACCGGCCAAAA	FAM - TGACCGGGATTTACGACCTACCAACCCT – TAMRA	CTTAAGACCCACCTATAG TGGCTACC	0.9/0.9/0.2	55	Chivero ET et al. The Journal of general virology. 2014;95:1307-19.
	Epstein-Barr virus	CGGAAGCCCTCTGGACT TC	FAM - TGTACACGCACGAGAAATGCGCCT – TAMRA	CCCTGTTTATCCGATGGA ATG	0.2/0.2/0.1	60	Kimura H et al. J Clin Microbiol 1999: 37:132-6
	Human herpes virus 7	Genesig™ Detection Kit, Human Herpesvirus 7			unknown	60	PrimerDesign, England, United Kingdom

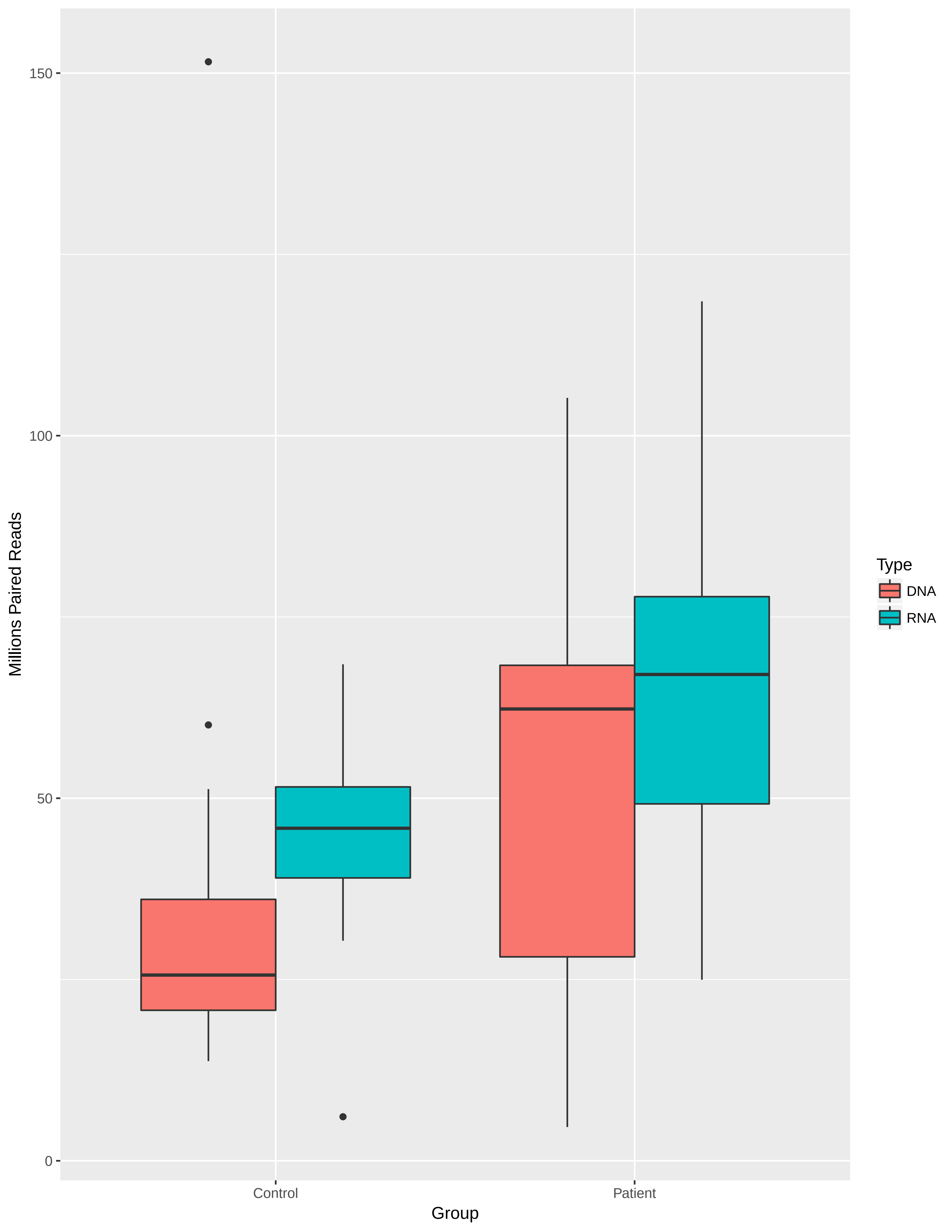

150
100
50
0
Millions Paired Reads
Control
Patient
Group
Type
DNA
RNA

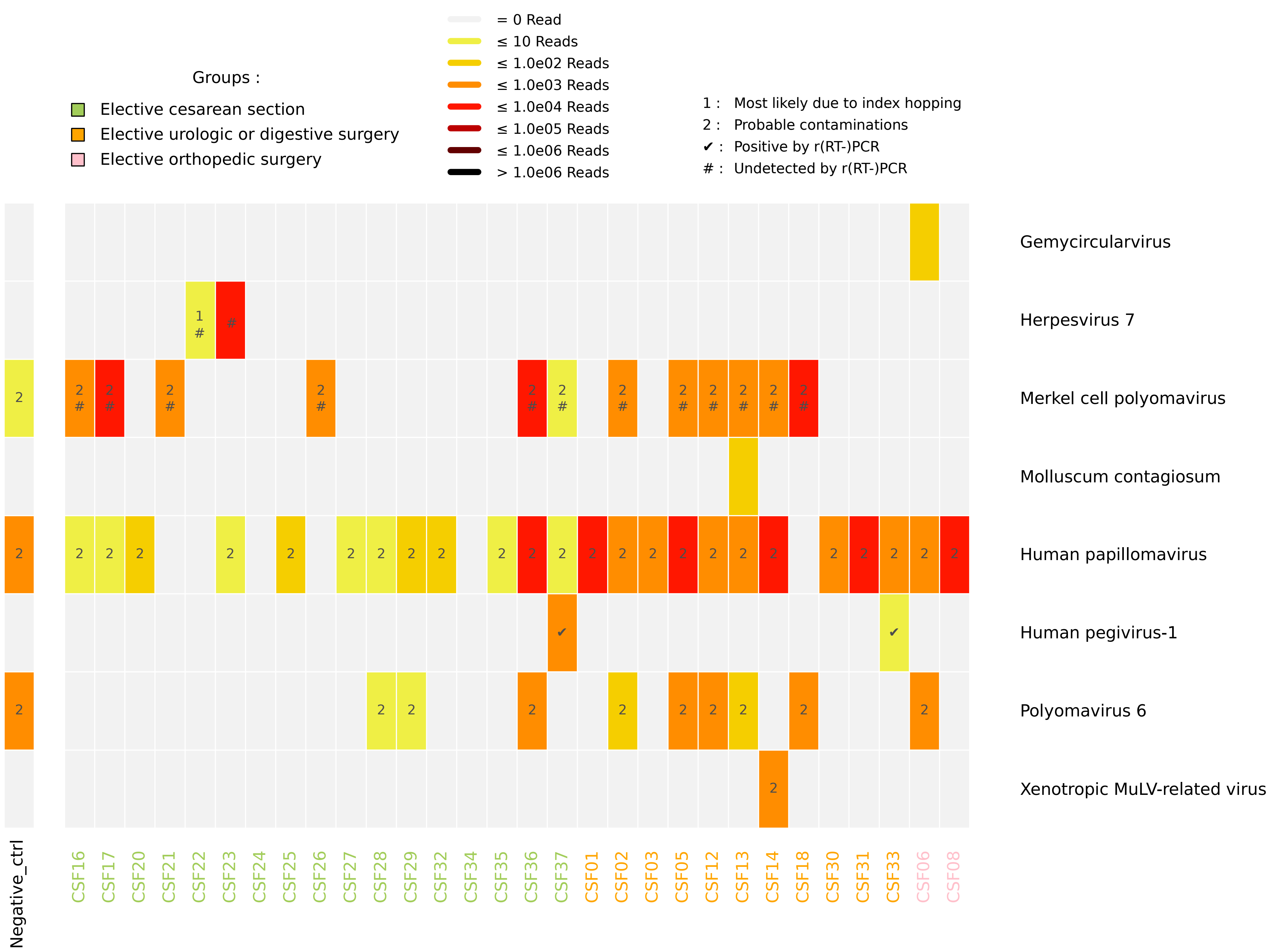

Groups :
Elective cesarean section
Elective urologic or digestive surgery
Elective orthopedic surgery
= 0 Read
≤ 10 Reads
≤ 1.0e02 Reads
≤ 1.0e03 Reads
≤ 1.0e04 Reads
≤ 1.0e05 Reads
≤ 1.0e06 Reads
> 1.0e06 Reads
1 : Most likely due to index hopping
2 : Probable contaminations
✔ : Positive by r(RT-)PCR
# : Undetected by r(RT-)PCR
Gemycircularvirus
Herpesvirus 7
Merkel cell polyomavirus
Molluscum contagiosum
Human papillomavirus
Human pegivirus-1
Polyomavirus 6
Xenotropic MuLV-related virus
Negative_ctrl
CSF16
CSF17
CSF20
CSF21
CSF22
CSF23
CSF24
CSF25
CSF26
CSF27
CSF28
CSF29
CSF32
CSF34
CSF35
CSF36
CSF37
CSF01
CSF02
CSF03
CSF05
CSF12
CSF13
CSF14
CSF18
CSF30
CSF31
CSF33
CSF06
CSF08

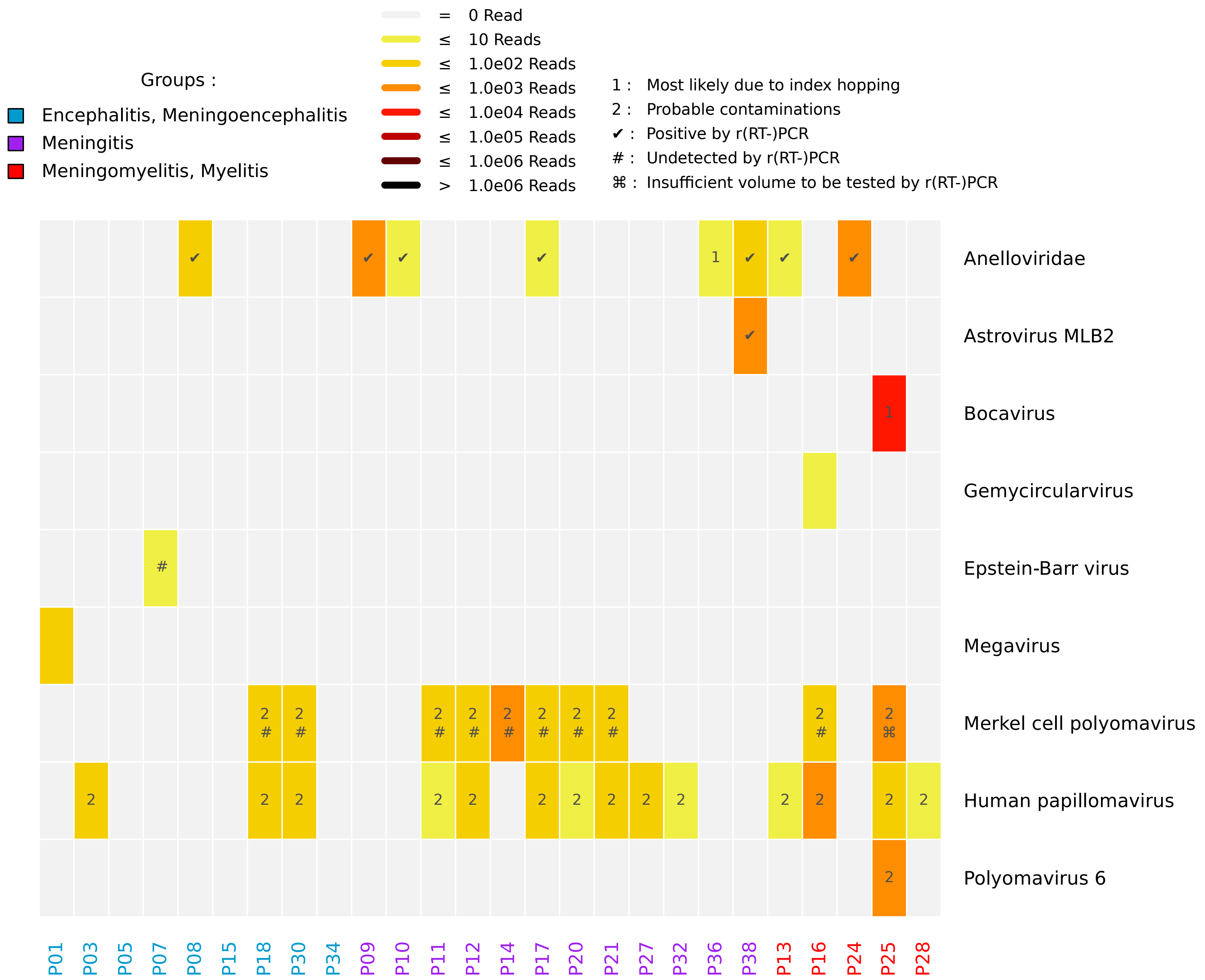

Groups :
Encephalitis, Meningoencephalitis
Meningitis
Meningomyelitis, Myelitis
= 0 Read
≤ 10 Reads
≤ 1.0e02 Reads
≤ 1.0e03 Reads
≤ 1.0e04 Reads
≤ 1.0e05 Reads
≤ 1.0e06 Reads
> 1.0e06 Reads
1 : Most likely due to index hopping
2 : Probable contaminations
✔ : Positive by r(RT-)PCR
# : Undetected by r(RT-)PCR
⌘ : Insufficient volume to be tested by r(RT-)PCR
Anelloviridae
Astrovirus MLB2
Bocavirus
Gemycircularvirus
Epstein-Barr virus
Megavirus
Merkel cell polyomavirus
Human papillomavirus
Polyomavirus 6
P01
P03
P05
P07
P08
P15
P18
P30
P34
P09
P10
P11
P12
P14
P17
P20
P21
P27
P32
P36
P38
P13
P16
P24
P25
P28

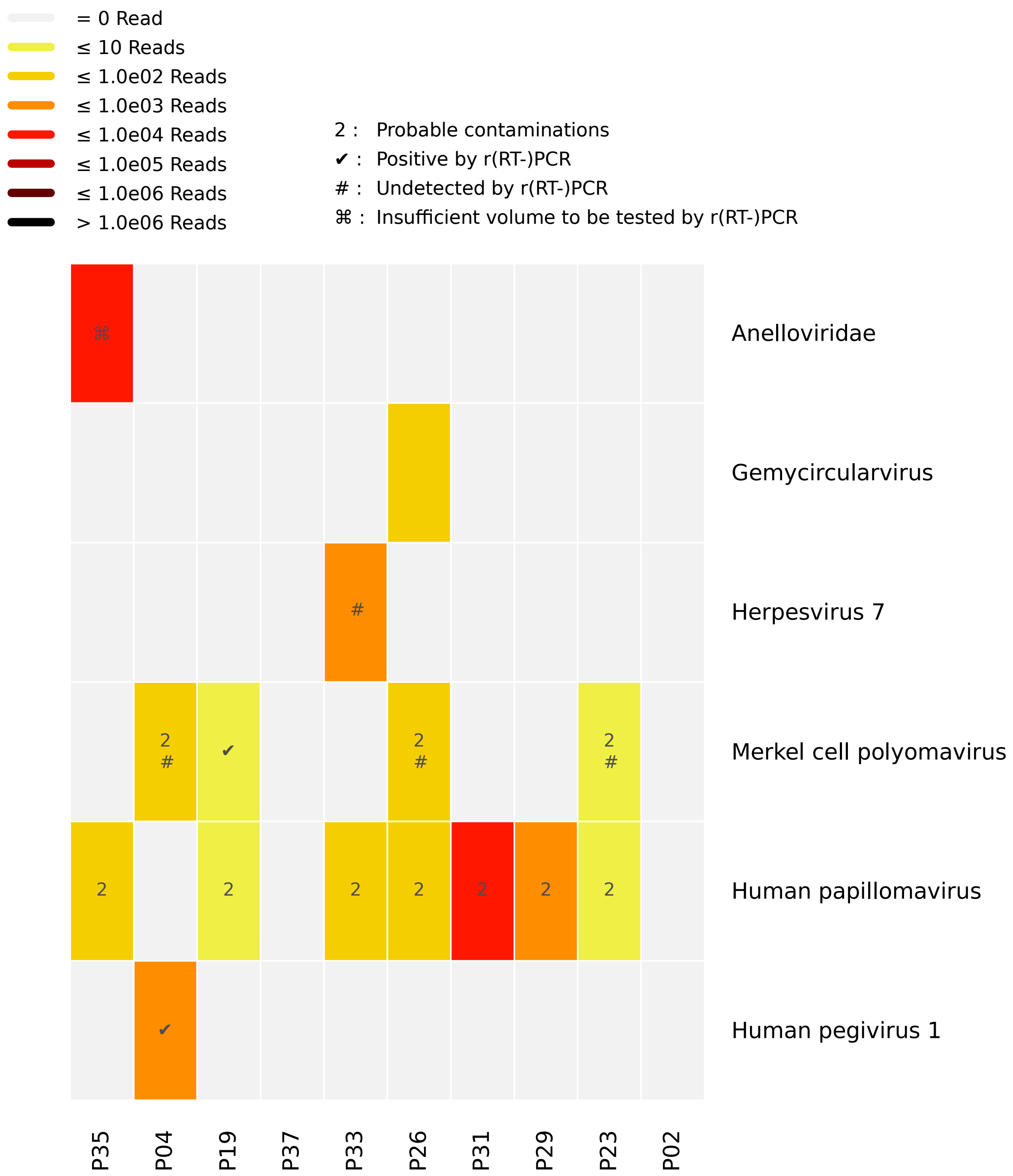

= 0 Read
≤ 10 Reads
≤ 1.0e02 Reads
≤ 1.0e03 Reads
≤ 1.0e04 Reads
≤ 1.0e05 Reads
≤ 1.0e06 Reads
> 1.0e06 Reads
2 : Probable contaminations
✔ : Positive by r(RT-)PCR
# : Undetected by r(RT-)PCR
⌘ : Insufficient volume to be tested by r(RT-)PCR
Anelloviridae
Gemycircularvirus
Herpesvirus 7
Merkel cell polyomavirus
Human papillomavirus
Human pegivirus 1
P35
P04
P19
P37
P33
P26
P31
P29
P23
P02
